# Multi-organ protection of ulinastatin in traumatic cardiac arrest model

**DOI:** 10.1186/s13017-018-0212-3

**Published:** 2018-11-12

**Authors:** Shaoyun Liu, Jiefeng Xu, Yuzhi Gao, Peng Shen, Senlin Xia, Zilong Li, Mao Zhang

**Affiliations:** 1 Department of Emergency Medicine, Second Affiliated Hospital, Zhejiang University School of Medicine, No. 88 Jiefang road, Hangzhou, 310009 China; 2 Institute of Emergency Medicine, Zhejiang University, No. 88 Jiefang road, Hangzhou, 310009 China; 3 Department of Emergency Medicine, Yuyao People’s Hospital, Medical School of Ningbo University, Yuyao, 315400 China; 4 Department of Emergency Medicine, The First Hospital of Jiaxing/The First Affiliated Hospital of Jiaxing University, Jiaxing, 314000 China; 5 Department of Emergency Medicine, Huzhou Central Hospital, Huzhou, 313000 China

**Keywords:** Ulinastatin, Traumatic cardiac arrest, Organ protection, Cardiopulmonary resuscitation, Post-resuscitation, Ischemia-reperfusion injury

## Abstract

**Background:**

Post-cardiac arrest syndrome, which has no specific curative treatment, contributes to the high mortality rate of victims who suffer traumatic cardiac arrest (TCA) and initially can be resuscitated. In the present study, we investigated the potential of ulinastatin to mitigate multiple organ injury after resuscitation in a swine TCA model.

**Methods:**

Twenty-one male pigs were subjected to hemodynamic shock (40% estimated blood loss in 20 min) followed by cardiac arrest (electrically induced ventricular fibrillation) and respiratory suspension for 5 min, and finally manual resuscitation. At 5 min after resuscitation, pigs were randomized to receive 80,000 U/kg ulinastatin (*n* = 7) or the same volume of saline (*n* = 9) in the TCA group. Pigs in the sham group (*n* = 5) were not exposed to bleeding or cardiac arrest. At baseline and at 1, 3, and 6 h after the return of spontaneous circulation, blood samples were collected and assayed for tumor necrosis factor-alpha, interleukin 6, and other indicators of organ injury. At 24 h after resuscitation, pigs were sacrificed and apoptosis levels were assessed in samples of heart, brain, kidney, and intestine.

**Results:**

One pig died in the ulinastatin group and one pig died in the TCA group; the remaining animals were included in the final analysis. TCA and resuscitation caused significant increases in multiple organ function biomarkers in serum, increases in tumor necrosis factor-alpha, and interleukin 6 in serum and increases in the extent of apoptosis in key organs. All these increases were lower in the ulinastatin group.

**Conclusion:**

Ulinastatin may attenuate multiple organ injury after TCA, which should be explored in clinical studies.

**Electronic supplementary material:**

The online version of this article (10.1186/s13017-018-0212-3) contains supplementary material, which is available to authorized users.

## Background

Traumatic cardiac arrest (TCA) is associated with poor outcome; traditionally, only 0–3.7% of TCA patients could be resuscitated [1], though advances in damage control resuscitation and understanding of TCA pathophysiology have increased the success rate to 5.6% (0–17%) [2], making it comparable to the rate with patients who suffer medical cardiac arrest [3]. Guidelines of the National Association of EMS Physicians and the Committee on Trauma of the American College of Surgeons do not recommend resuscitation for patients who suffer blunt trauma and out-of-hospital cardiac arrest and found pulseless, without organized ECG activity, or for patients who have suffered penetrating trauma and have no detectable pulse and signs of life [4]. TCA is associated with greater loss of productive life years than medical cardiac arrest because TCA usually affects young men [3]. More than 90% of TCA cases are caused by severe head injury or hypovolemia [4]. Even among TCA patients who achieve a return of spontaneous circulation (ROSC), mortality rates are high because of the prolonged whole-body ischemia during trauma and cardiac arrest and because of reperfusion injury after resuscitation.

It may be possible to reduce TCA-induced damage using the urinary trypsin inhibitor (UTI) ulinastatin, which was first identified in human blood and urine in the 1980s. Ulinastatin, which inhibits the release of neutrophil elastase and inhibits the activation of various pro-inflammatory cytokines [3, 5], is used mainly to treat pancreatitis, sepsis, toxic shock, and hemorrhagic shock [5, 6]. In animal models of brain ischemia-reperfusion, ulinastatin decreased infarct volume and water content of brain tissue, and it inhibited cerebral apoptosis, thereby mitigating ischemia-reperfusion injury [7, 8]. A meta-analysis of randomized controlled trials concluded that ulinastatin can protect pulmonary tissue for patients undergoing cardiac surgery and reduce postoperative increases in the inflammatory agent’s tumor necrosis factor (TNF)-alpha, polymorphonuclear neutrophil elastase, and interleukin (IL)-6 and IL-8 [9]. In addition, ulinastatin may be associated with a low incidence of acute kidney injury in patients undergoing robot-assisted laparoscopic partial nephrectomy or cardiac surgery [10, 11].

Few studies have examined the efficacy and safety of ulinastatin in patients who have suffered cardiac arrest, particularly TCA. In the present study, we examined whether ulinastatin can mitigate the effects of TCA and ischemia-reperfusion based on serum indicators and extent of apoptosis in major organs in a porcine TCA model.

## Methods

The experimental procedures used in this study adhered to the US National Institutes of Health Guide for the Care and Use of Laboratory Animals.

### TCA

Twenty-one male Chinese pigs (29–36 kg) were used for this study. Anesthesia was induced using an intramuscular injection of ketamine (500 mg) and midazolam (5 mg) and maintained with propofol (3 mg/kg/h) and fentanyl (1 μg/kg/h) during surgical procedures. Respiration was maintained using artificial ventilation (SynoVent E5; Mindray Biomedical Electronics, Shenzhen, China) with the following settings: respiratory rate, 12–15 bpm; inspired oxygen, 21% and PEEP, 3 mmHg. A catheter was inserted into the aortic artery through the left femoral artery and another catheter was inserted into the right atrium through the left jugular vein. Both catheters were connected to pressure transducers. Heart rate (HR), mean aortic blood pressure (MAP), and right atrial pressure (RAP) were monitored (Beneview T6; Mindray Biomedical Electronics, Shenzhen, China). The cannula in the right femoral artery was used for controlled blood-letting 40% of estimated blood volume at a constant rate during 20 min with a blood pump (JHBP-2000B; JIHUA Medical Apparatus & Instruments, Guangzhou, China). Then, ventricular fibrillation was induced using a modified voltmeter (85 L1-A; Yongsheng Electric Instrument Co., Guangzhou, China). Respiration was suspended by separating the tracheal cannula from the ventilator. Artificial ventilation was resumed 5 min later, and pigs were defibrillated (Zoll Medical Corporation, USA) and manually resuscitated. Pigs that achieved ROSC were used in further interventions.

### Experimental procedures

At 5 min after ROSC, pigs were infused with ulinastatin (80,000 U/kg; *n* = 6) or the same volume of normal saline (TCA group; *n* = 8) into the right femoral vein during 2 min. Half the blood withdrawn from the right femoral artery was reinfused through the right femoral vein during the first hour after ROSC. During the next 1-h period, pigs received a volume of normal saline equal to three times the volume of blood lost. As an additional control, sham pigs (*n* = 5) underwent surgery but were not subjected to bleeding, cardiac arrest or resuscitation.

### Outcome measures

HR, MAP, RAP, and end-tidal carbon dioxide (ETCO_2_) were recorded at baseline and at 1, 3, and 6 h after ROSC. Blood gases and coagulation function were also analyzed at these time points. Plasma samples stored at − 80 °C were assayed for creatine kinase MB (CK-MB), cardiac troponin I (cTNI), neuron-specific enolase (NSE), S100 calcium-binding protein B (S100B), serum creatinine (sCr), blood urea nitrogen (BUN), intestinal fatty acid-binding protein (iFABP), diamine oxidase (DAO), IL-6, and TNF-α.

Animals were sacrificed at 24 h after ROSC. Samples of the apical myocardium, frontal cortex of the brain, infrarenal pole cortex, and terminal ileum were removed and stored at − 80 °C. Then, the samples were assessed for the extent of apoptosis using terminal deoxynucleotidyl transferase-mediated dUTP nick end labeling (TUNEL), and they were analyzed for caspase-3 expression using immunohistochemistry.

### Statistical analysis

Continuous data are presented as mean ± standard deviation (SD) and inter-group pairwise differences were analyzed using repeated measures one-way analysis of variance, followed by parametric Student’s *t* test. All statistical analyses were performed using SPSS for Windows 20.0 (IBM, Chicago, IL, USA), and statistical significance was defined as *p* < 0.05.

## Results

### Baseline characteristics

The three groups were similar at baseline in terms of body weight, HR, MAP, and, ETCO_2_ (all *p* > 0.05; Table 1). One pig died in the ulinastatin group and one in the TCA group. Additionally, all resuscitated animals survived to the end of the experiment.

**Table 1:** Baseline characteristics^a^

Characteristic	Sham (*n* = 5)	TCA (*n* = 9)	Ulinastatin (*n* = 7)	*p* ^b^
Body weight (kg)	35.00 ± 1.41	31.75 ± 3.99	31.67 ± 1.51	0.119
Heart rate (bpm)	86 ± 8	85 ± 15	85 ± 13	0.978
ETCO_2_/mmHg	40 ± 2	39 ± 2	39 ± 2	0.623
MAP (mmHg)	117 ± 5	104 ± 16	106 ± 13	0.221
Death/survival	0/5	1/8	1/6	0.725

^a^mean ± SD. *ETCO*_*2*_ end-tidal carbon dioxide, *MAP* mean aortic blood pressure, *TCA* traumatic cardiac arrest

^b^There were no significant differences among the groups (one-way analysis of variance)

### Hemodynamics

**Fig. 1:**
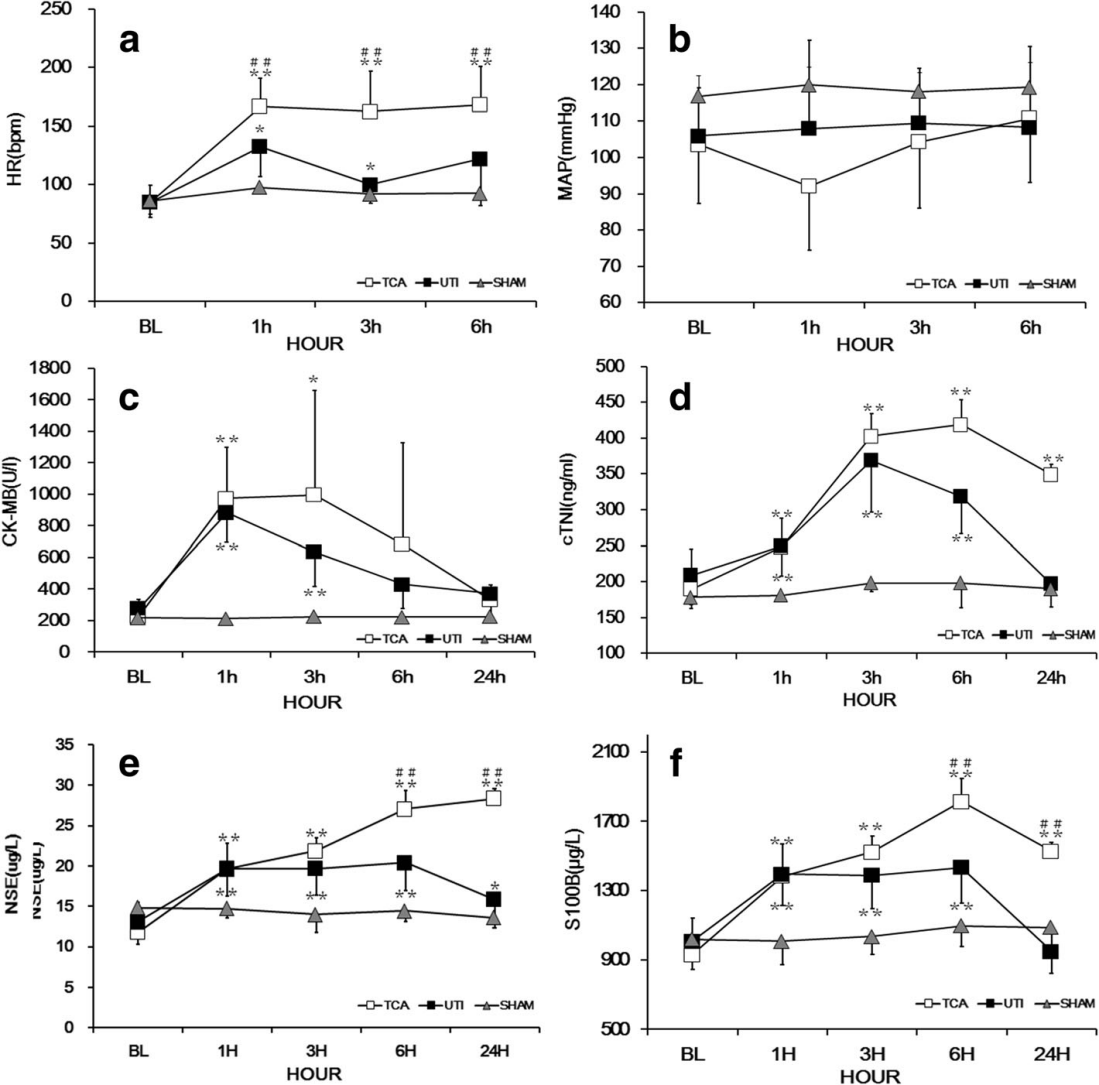
Hemodynamics and biomarkers of myocardial or cerebral injury among the three groups. **a** Heart rate (HR). **b** Mean aortic pressure (MAP). **c** Creatine kinase MB (CK-MB). **d** Cardiac troponin I (cTNI). **e** Neuron-specific enolase (NSE). **f** S100 calcium-binding protein B (S100B). The x-axis is the time point of baseline 1, 3, 6, and 24 h after ROSC. Values shown are mean ± SD. TCA, traumatic cardiac arrest; UTI, ulinastatin; BL, baseline; ROSC, return of spontaneous circulation. *p < 0.05, **p < 0.01 vs. baseline; #*p* < 0.05, ##*p* < 0.01 vs. ulinastatin

After ROSC, HR was significantly higher in the TCA group than in the sham group (*p* < 0.05; Fig. 1a). HR was significantly lower in the ulinastatin group than in the TCA group (*p* < 0.05). MAP after ROSC did not differ significantly among the three groups (*p* > 0.05; Fig. 1b).

### Serum biomarkers of myocardial injury

Prior to surgery, neither serum CKMB nor cTNI differed significantly among the three groups (all *p* > 0.05; Fig. 1c, d). CKMB increased from the pre-operative baseline and peaked at 1 h after ROSC in the ulinastatin group or at 3 h after ROSC in the TCA group. Similarly, cTNI increased from baseline and peaked at 3 h after ROSC in the ulinastatin group or at 6 h in the TCA group. Serum CK-MB and cTNI in the TCA group were significantly higher after ROSC than in the sham group (*p* < 0.01). CKMB after ROSC did not differ significantly between the TCA and ulinastatin groups (*p* > 0.05). The level of cTNI after ROSC was lower in the ulinastatin group than in the TCA group (*p* < 0.01).

### Serum biomarkers of cerebral injury

Serum concentration of NSE and S100B did not differ among the three groups at baseline (all *p* > 0.05; Fig. 1e, f). Levels of both biomarkers were significantly higher after ROSC than at baseline in the TCA and ulinastatin groups (both *p* < 0.01). Levels of both biomarkers after ROSC were higher in the TCA group than in the sham control (both *p* < 0.01). The levels of both biomarkers after ROSC in the ulinastatin group were significantly lower than those in the TCA group (both *p* < 0.01).

### Serum biomarkers of renal injury

**Fig. 2:**
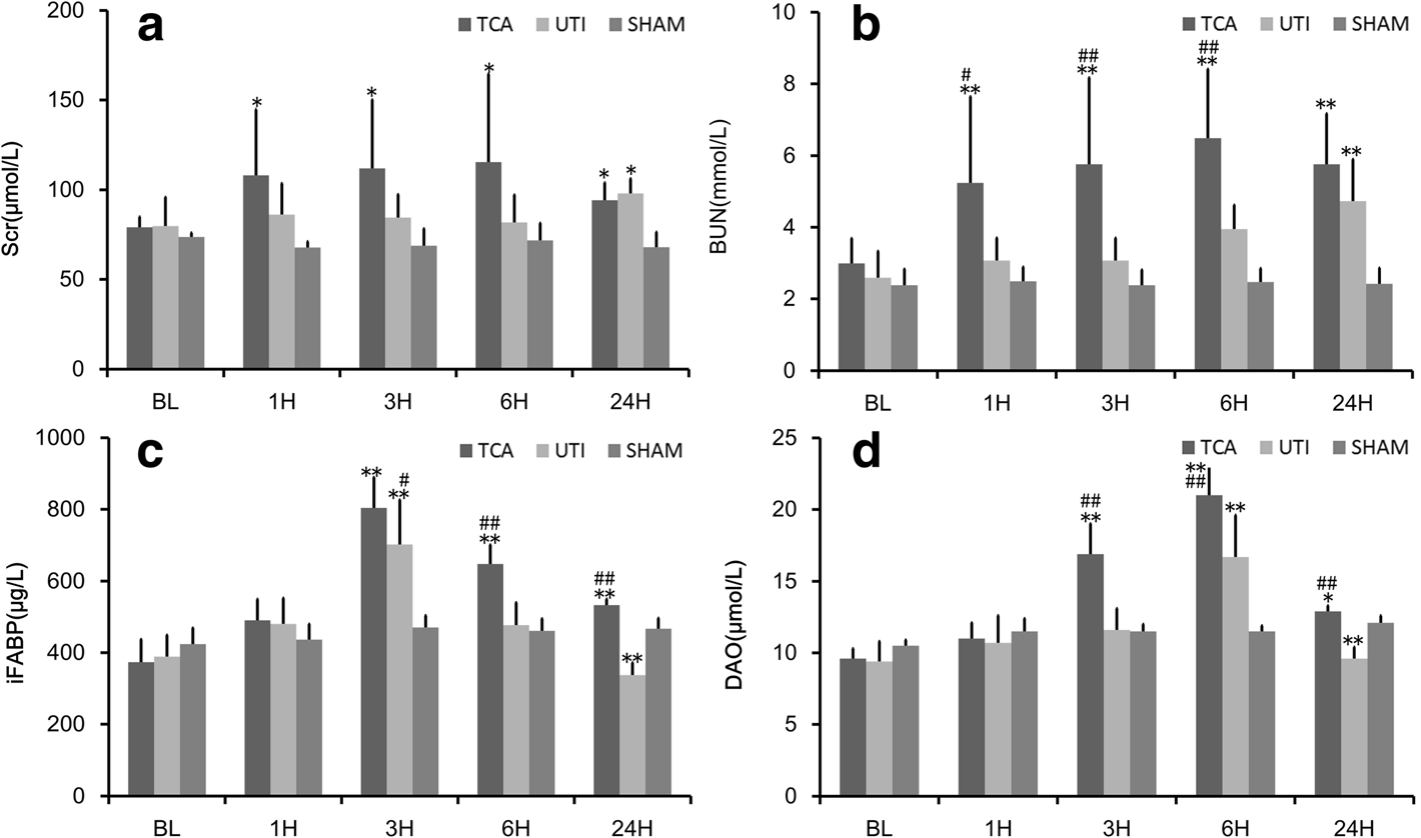
Biomarkers of intestinal or renal injury in the three groups. **a** Serum creatinine (sCr). **b** Blood urea nitrogen (BUN). **c** Intestinal fatty acid-binding protein (iFABP). **d** Diamine oxidase (DAO). The x-axis is the time point of baseline 1, 3, 6, and 24 h after ROSC. Values shown are mean ± SD. TCA, traumatic cardiac arrest; UTI, ulinastatin; BL, baseline; ROSC, return of spontaneous circulation. *p < 0.05, **p < 0.01 vs. baseline; #*p* < 0.05, ##*p* < 0.01 vs. ulinastatin

At baseline, sCr and BUN did not differ among the three groups (both *p* > 0.05; Fig. 2a, b). The level of sCr after ROSC was similar among the three groups (all *p* > 0.05). BUN after ROSC was significantly higher in the ulinastatin group than at baseline (*p* < 0.01), whereas BUN was similar between the ulinastatin and sham groups (*p* > 0.05). BUN after ROSC was lower in the ulinastatin group than in the TCA group (*p* < 0.01).

### Serum biomarkers of intestinal injury

At baseline, serum concentrations of NSE and S100B did not differ among the three groups (all *p* > 0.05; Fig. 2c, d). After ROSC, the levels of iFABP and DAO were significantly higher in the TCA group than in the sham group (both *p* < 0.01) and significantly lower in the ulinastatin group than in the TCA group (both *p* < 0.05).

### Arterial blood gas analysis

Baseline PaO_2_, SaO_2,_ pH, and lactate concentration were not significantly different among the three groups (all *p* > 0.05; Additional file 1: Table S1). PaO_2_ and SaO_2_ of arterial blood after ROSC were higher in the ulinastatin group than in the TCA group (both *p* < 0.05). The pH after ROSC was lower in the TCA group than in the sham group (*p* < 0.05), and it tended to be higher in the ulinastatin group than in the TCA group, although the difference did not achieve statistical significance (*p* > 0.05). Lactate after ROSC was significantly higher in the TCA group than in the sham group (*p* < 0.01), while lactate was significantly lower in the ulinastatin group than in the TCA group (*p* < 0.05).

### Coagulation functions

Baseline PT, INR, APTT, and serum level of fibrinogen did not differ significantly among the three groups (all *p* > 0.05; Additional file 1: Table S2). PT, INR, and FIB after ROSC did not differ significantly among the three groups (all *p* > 0.05). APTT was significantly higher in the ulinastatin group than in the TCA group (*p* < 0.05), whereas it was similar between the TCA and sham groups (*p* > 0.05).

### Serum TNF-α and IL-6

**Fig. 3:**
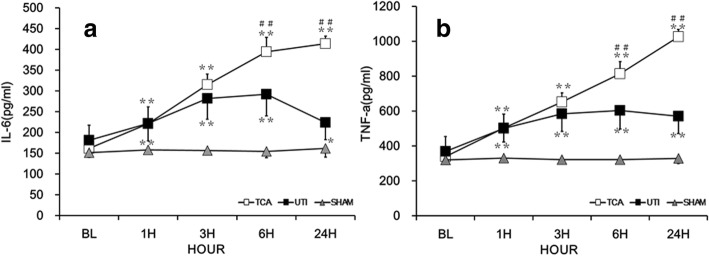
IL-6 and TNF-α among the three groups. **a** Interleukin (IL)-6. **b** Tumor necrosis factor (TNF)-α. The x-axis is the time point of baseline 1, 3, 6, and 24 h after ROSC. Values shown are mean ± SD. TCA, traumatic cardiac arrest; UTI, ulinastatin; BL, baseline; ROSC, return of spontaneous circulation. *p < 0.05, **p < 0.01 vs. baseline; #*p* < 0.05, ##*p* < 0.01 vs. ulinastatin

Preoperative TNF-α and IL-6 did not differ significantly among the three groups (all *p* > 0.05; Fig. 3a, b). Serum TNF-α and IL-6 in the TCA group were significantly higher after ROSC than in the sham group (both *p* < 0.01). Serum TNF-α and IL-6 were significantly lower after ROSC in ulinastatin group than in the TCA group (both *p* < 0.01).

### Histopathology of major organs

**Fig. 4:**
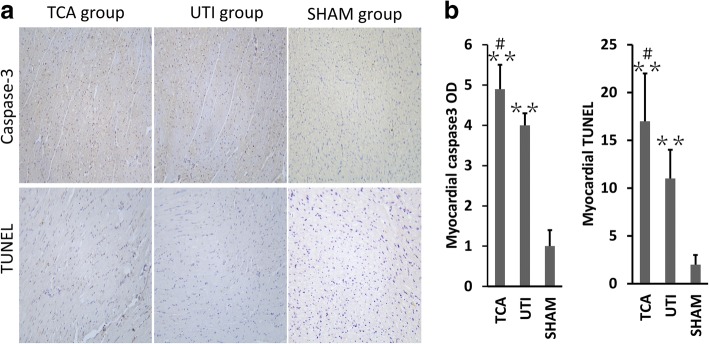
Ulinastatin-mediated inhibition of apoptosis in the heart. **a** Anti-caspase-3 immunostaining and TUNEL assay results in the three groups. **b** Optical density of caspase-3 immunostaining and numbers of TUNEL-positive cells in the three groups. TCA, traumatic cardiac arrest; UTI, ulinastatin; BL, baseline; TUNEL, terminal deoxynucleotidyl transferase-mediated dUTP nick end labeling. *p < 0.05, **p < 0.01 vs. sham; #*p* < 0.05, ##*p* < 0.01 vs. ulinastatin

The optical density of immunostaining against caspase-3 as well as the number of TUNEL-positive cells in the apical myocardium were significantly higher in the TCA group than in the sham group (both *p* < 0.01; Fig. 4a, b), while both of these parameters were lower in the ulinastatin group than in the TCA control (both *p* < 0.05).

**Fig. 5:**
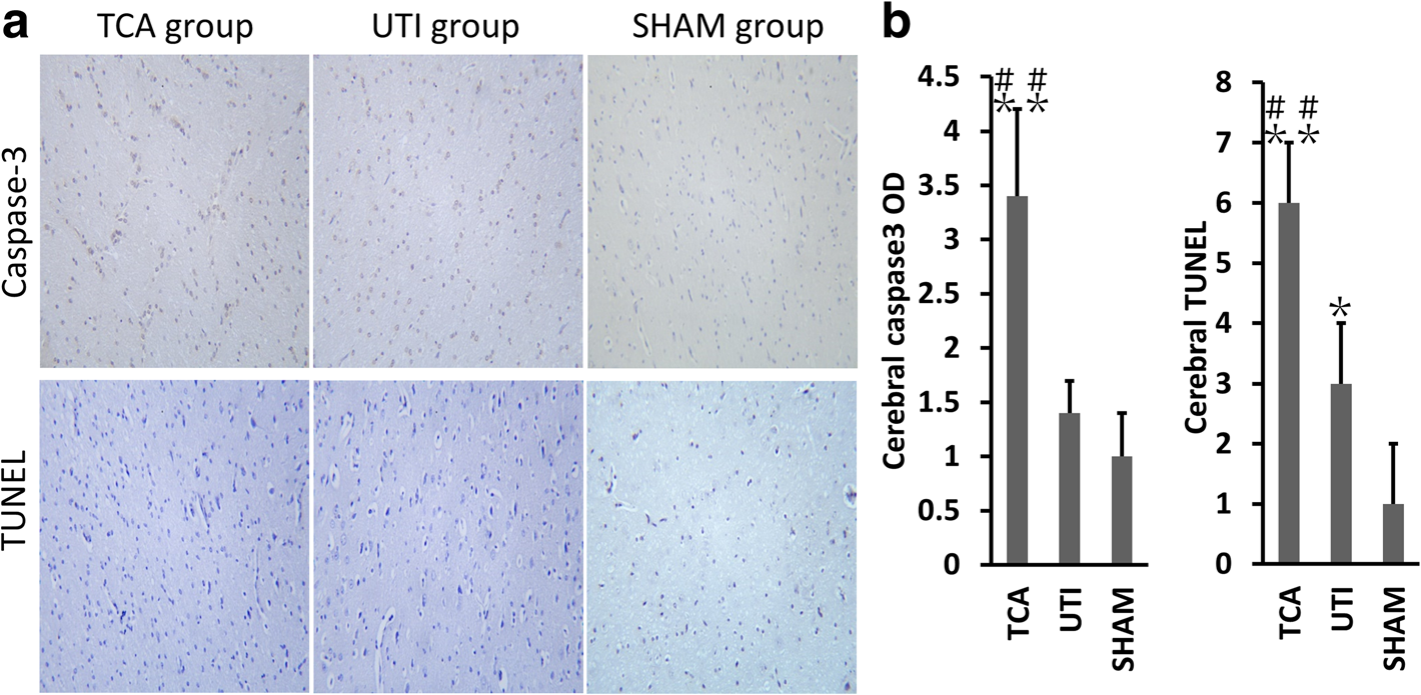
Ulinastatin-mediated inhibition of apoptosis in cerebral tissue. **a** Anti-caspase-3 immunostaining and TUNEL assay results in the three groups. **b** Optical density of caspase-3 immunostaining and numbers of TUNEL-positive cells in the three groups. TCA, traumatic cardiac arrest; UTI, ulinastatin; BL, baseline; TUNEL, terminal deoxynucleotidyl transferase-mediated dUTP nick end labeling. *p < 0.05, **p < 0.01 vs. sham; #*p* < 0.05, ##*p* < 0.01 vs. ulinastatin

The optical density of caspase-3 immunostaining in cerebral frontal tissue was significantly higher in the TCA group than in the sham group (*p* < 0.01; Fig. 5a, b), while it was significantly lower in the ulinastatin group than in the TCA group (*p* < 0.01). The same results were observed for the number of TUNEL-positive cells (*p* < 0.05 and *p* < 0.01, respectively).

The optical density of caspase-3 immunostaining and the number of TUNEL-positive cells in the renal cortex were significantly higher in the TCA group than in the sham group (both *p* < 0.01; Additional file 1: Table S3). Ulinastatin attenuated both measures of apoptosis (both *p* < 0.01 vs. TCA group).

The optical density of caspase-3 immunochemistry in terminal ileum was significantly higher in the TCA group than in the sham group (*p* < 0.05; Additional file 1: Table S3), whereas it was similar between the TCA and ulinastatin groups (*p* > 0.05). The number of TUNEL-positive cells was significantly higher in the TCA group than in the sham group (*p* < 0.01) and significantly lower in the ulinastatin group than in the TCA group (*p* < 0.01).

## Discussion

The present study suggests that ulinastatin can improve hemodynamics and attenuate multiple organ injury following TCA in a porcine model. After ROSC, administration of ulinastatin suppressed the increase in HR and biomarkers of major organ injury. Ulinastatin was associated with significantly higher PaO_2_ and SaO_2_ in arterial blood, as well as lower lactic acid, than in the TCA group. TCA in the present study was induced by controlled bleeding of 40% of total blood volume, followed by ventricular fibrillation. This model faithfully simulates the pathological causes of TCA in humans.

The main pathophysiological feature of TCA is ischemia-reperfusion injury [12], which is the main driver of morbidity and mortality in TCA patients. Ischemic damage occurs immediately during hemorrhage and cardiac arrest, whereas reperfusion injury occurs after ROSC. Such injury is associated with increases in oxygen free radicals and inflammatory factors, for example, superoxide anion free radical, hydrogen peroxide, hydroxyl radical, and TNF-α [7, 13–15]. In both animal studies and randomized clinical trials in humans, ulinastatin has shown protective effects against ischemia-reperfusion injury, leading to lower incidence of organ injury and higher survival rates [6, 9, 16–18]. How ulinastatin can mitigate ischemia-reperfusion injury remains unclear.

The present study showed that ulinastatin suppressed the increase in serum inflammatory factors after ROSC, consistent with previous studies of sepsis and septic shock [5, 6]. Inflammation plays an important role in ischemia-reperfusion injury and in apoptosis induction in multiple organs. Ulinastatin downregulates Toll-like receptors (TLRs) and NF-κB expression and protects the brain against ischemia-reperfusion injury [19]. TLRs form a complex with MyD88 to activate inflammatory cytokines and NF-κB, which regulates the expression of a wide array of genes involved in immune responses [19]. In the present study, we found that ulinastatin reduced TNF-α expression and IL-6 upregulation, consistent with previous studies [12, 20]. TNF-α can stimulate autophagy, and IL-13 suppresses autophagy by stimulating the phosphoinositide 3-kinase/mTOR signal transduction pathway [20]. In cell culture, ulinastatin may downregulates the autophagy marker LC3-II by improving cell viability in the face of hypoxia/deoxygenation [20]. Ulinastatin also reverses the upregulation of water transporter aquaporin 4 induced in heart and brain tissue in response to cerebral hemorrhage, cerebral trauma or cardiopulmonary resuscitation, and ulinastatin mitigating damage to cardiac arrest function by decreasing the expression of aquaporin 4 [21]. These various studies suggest that ulinastatin may help protect against ischemia injury by inhibiting proteases, inflammatory responses, and cytokine-dependent signaling pathways.

In the present study, the extent of myocardial injury was assessed based on CK-MB and C-TNI; the extent of intestinal injury, based on iFABP and DAO; and the extent of brain injury, NSE and S-100B, which are the most commonly used blood markers of such injury. Elevated NSE and S-100B are associated with poor outcome [22].

In the present study, ulinastatin attenuated tissue injury and cell apoptosis in the brain, heart, kidneys, and intestine. Ulinastatin appears to attenuate ischemia-reperfusion injury in multiple organs by suppressing inflammation and oxidative stress [7, 11, 23], which reduces the generation of oxygen free radicals that accompanies many pathological states such as inflammation, ischemia, and reperfusion [12]. Ischemia-reperfusion injury can cause endothelial barrier dysfunction, resulting in high vascular permeability and tissue edema, and ulinastatin has been shown to inhibit vascular hyperpermeability [24, 25]. Tissue edema reduces the supply of oxygen and nutrients, as well as the removal of waste products from tissues. The ischemia-reperfusion injury also triggers endothelial cell inflammation that results in vascular dysfunction [8, 12]. Crystalloids have several disadvantages with respect to blood infusion: they improve circulation but cannot carry oxygen, they require the infusion of a greater volume of fluid, and they are associated with worse interstitial edema [4]. Ischemia-reperfusion injury induces endothelial production of vasoactive substances that cause vasoconstriction [12].

Factors that contribute to ischemia-reperfusion injury include energy metabolism, changes in the mitochondria and cellular membranes, initiation of different forms of cell death-like apoptosis, and necrosis [12, 26]. Energy metabolism depends on the delivery of blood and oxygen to the tissue, and it depends on the overall metabolic activity in the tissue. After ROSC, HR rises to increase stroke volume and maintain arterial blood pressure in order to compensate for hypovolemia. This increases ATP consumption and energy demand in the myocardium. In the present study, ulinastatin suppressed the increase in HR while maintaining aortic blood pressure. Ischemia-reperfusion initiates different forms of cell death-like apoptosis and necrosis, which recruits inflammatory cells to the necrotic areas and stimulates the release of cytokines [12]. Here, we showed that ulinastatin decreased apoptosis in major organs.

There are some limitations in the present study. First, cardiac arrest was induced by an electrode to produce ventricular fibrillation. However, ventricular arrhythmia occurs in < 3% of TCA patients, and 30–60% of patients present with pulseless electrical activity [4]. Second, ulinastatin in the present study was given at 80000 U/kg at 5 min after ROSC, but this regime may not be optimal for patients with TCA. Third, the observation period was only 24 h after ROSC, which is too short to capture the entire spectrum of post-cardiac arrest syndrome.

## Conclusions

Ulinastatin can improve hemodynamics and attenuate multiple organ injury after TCA in a large animal model. Further clinical studies are needed.

## Additional file

Additional file 1: **Table S1.** Blood gas analysis over time after ROSC in the TCA group (*n* = 8), ulinastatin group (*n* = 6) and sham group (*n* = 5). Table S2. Coagulation function over time after ROSC in the TCA group (*n* = 8), ulinastatin group (*n* = 6) and sham group (*n* = 5). Table S3. Caspase-3 levels and numbers of TUNEL-positive cells in the heart, cerebral, lung, renal, and intestinal tissues of the three groups. (DOCX 57 kb)

## Abbreviations

BUN: Blood urea nitrogen
CK-MB: Creatine kinase MB
cTNI: Cardiac troponin I
DAO: Diamine oxidase
ETCO2: End-tidal carbon dioxide
HR: Heart rate
iFABP: Intestinal fatty acid-binding protein
IL: Interleukin
MAP: Mean aortic blood pressure
NSE: Neuron-specific enolase
RAP: Right atrial pressure
ROSC: Return of spontaneous circulation
S100B: S100 calcium-binding protein B
sCr: Serum creatinine
SD: Standard deviation
TCA: Traumatic cardiac arrest
TNF: Tumor necrosis factor
TUNEL: Terminal deoxynucleotidyl transferase-mediated dUTP nick end labeling
UTI: Urinary trypsin inhibitor
