# Supplementary material for: Multi-organ protection of ulinastatin in traumatic cardiac arrest model
Source: World J Emerg Surg. 2018 Nov 12;13:51. doi: 10.1186/s13017-018-0212-3 (PMC6233498; doi:10.1186/s13017-018-0212-3)
Supplement: Supplementary file 1 — Table S1. Blood gas analysis over time after ROSC in the TCA group (n = 8), ulinastatin group (n = 6) and sham group (n = 5). Table S2. Coagulation function over time after ROSC in the TCA group (n = 8), ulinastatin group (n = 6) and sham group (n = 5). Table S3. Caspase-3 levels and numbers of TUNEL-positive cells in the heart, cerebral, lung, renal, and intestinal tissues of the three groups. (DOCX 57 kb) [file 13017_2018_212_MOESM1_ESM.docx]

| **Additional file 1**  **Table S1.** Blood gas analysis and coagulation over time after ROSC in the TCA group (n=8), ulinastatin group (n=6) and sham group (n=5) | | | | |
| --- | --- | --- | --- | --- |
| Parameter and group | Baseline | h after ROSC | | |
|  |  | 1 | 3 | 6 |
| **PaO_2_ (mmHg)** | |  |  |  |
| TCA | 91±13 | 83±11 | 87±8^＃＃^ | 83±11^＃^ |
| Ulinastatin | 105±13 | 92±18 | 100±5 | 96±10 |
| Sham | 92±12 | 94±8 | 89±10 | 84±5 |
| **SaO_2_ (%)** | |  |  |  |
| TCA | 97±1 | 91±5 | 96±1^＃＃^ | 96±2^＃^ |
| Ulinastatin | 98±1 | 95±3^**^ | 98±0 | 98±1 |
| Sham | 96±4 | 98±1 | 98±2 | 97±1 |
| **LAC (mmol/L)** | |  |  |  |
| TCA | 2.2±2.2 | 9.5±3.2^**＃^ | 6.8±4.0^**＃^ | 3.5±3.0 |
| Ulinastatin | 1.5±0.6 | 5.7±1.7^**^ | 1.9±1.1 | 1.5±1.1 |
| Sham | 1.3±0.7 | 0.6±0.3 | 0.8±0.3 | 0.6±0.2 |
| **pH** |  |  |  |  |
| TCA | 7.47±0.06 | 7.18±0.18^**^ | 7.39±0.10^*^ | 7.47±0.09 |
| Ulinastatin | 7.50±0.03 | 7.32±0.08^**^ | 7.50±0.04 | 7.51±0.05 |
| Sham | 7.46±0.95 | 7.47±0.04 | 7.47±0.03 | 7.47±0.04 |

TCA, traumatic cardiac arrest; LAC, lactic acid; ROSC, return of spontaneous circulation.

Values are mean±SD. **p* < 0.05, ***p* < 0.01 vs. baseline; #*p* < 0.05, ##*p* < 0.01 vs. ulinastatin.

**Table S2.** Coagulation function over time after ROSC in the TCA group (n=8), ulinastatin group (n=6) and sham group (n=5)

| Functional parameter and group | Baseline | h after ROSC | | |
| --- | --- | --- | --- | --- |
|  |  | 1 | 3 | 6 |
| **PT** |  |  |  |  |
| TCA | 12.8±0.3 | 13.9±0.9 | 13.1±0.6 | 13.5±0.6 |
| Ulinastatin | 13.4±1.1 | 13.5±0.7 | 13.1±0.4 | 13.2±0.5 |
| Sham | 13.2±0.4 | 13.5±0.2 | 13.6±0.1 | 13.7±0.1 |
| **INR** |  |  |  |  |
| TCA | 0.98±0.02 | 1.09±0.09 | 1.01±0.06 | 1.05±0.05 |
| Ulinastatin | 1.04±0.11 | 1.05±0.07 | 1.01±0.03 | 1.03±0.04 |
| Sham | 1.05±0.01 | 1.07±0.03 | 1.08±0.12 | 1.09±0.07 |
| **APTT** |  |  |  |  |
| TCA | 37.9±10.6 | 67.0±48.7^##^ | 29.5±7.6^##^ | 38.9±4.8^#^ |
| Ulinastatin | 45.1±9.0 | 96.5±63.2 | 51.1±11.2 | 50.5±9.3 |
| Sham | 37.0±4.8 | 37.9±5.2 | 39.3±5.4 | 39.4±1. 6 |
| **FIB** |  |  |  |  |
| TCA | 2.34±1.28 | 1.67±0.83 | 1.84±0.84 | 1.86±0.94 |
| Ulinastatin | 2.10±0.27 | 1.49±0.28 | 1.77±0.25 | 1.86±0.29 |
| Sham | 2.10±0.31 | 2.09±0.36 | 2.14±0.34 | 2.30±0.35 |

PT, prothrombin time; INR, international normalized ratio; FIB, fibrinogen; APTT, activated partial thromboplastin time; ROSC, return of spontaneous circulation; TCA, traumatic cardiac arrest.

Values are mean±SD. **p* < 0.05, ***p* < 0.01 vs. sham; #*p* < 0.05, ##*p* < 0.01 vs. ulinastatin.

| **Table S3.** Caspase-3 levels and numbers of TUNEL-positive cells in heart, cerebral, lung, renal, and intestinal tissues of the three groups | | | |
| --- | --- | --- | --- |
| Parameter and tissue | TCA  (n=8) | Ulinastatin  (n=6) | Sham  (n=5) |
| **Optical density of anti-caspase-3 immunostaining** | | | |
| Heart | 4.9±0.6^**＃^ | 4.0±0.3^**^ | 1.0±0.4 |
| Brain | 3.4±0.8^**＃＃^ | 1.4±0.3 | 1.0±0.4 |
| Lung | 3.4±0.8^**＃＃^ | 1.5±0.3 | 1.9±0.6 |
| Kidney | 5.2±0.6^**＃＃^ | 2.6±0.8^**^ | 0.8±0.2 |
| Intestine | 5.5±2.6^*^ | 3.0±0.9 | 3.8±2.2 |
| **Number of TUNEL-positive cells** | | | |
| Heart | 17±5^**＃^ | 11±3^**^ | 2±1 |
| Brain | 6±1^**＃＃^ | 3±1^*^ | 1±1 |
| Lung | 12±3^**＃＃^ | 8±3^**^ | 1±1 |
| Kidney | 15±2^**＃＃^ | 9±4^**^ | 1±1 |
| Intestine | 17±2^**＃＃^ | 9±1^**^ | 1±1 |

Values are mean±SD. TCA, traumatic cardiac arrest; TUNEL, terminal deoxynucleotidyl transferase-mediated dUTP nick end-labeling. **p* < 0.05, ***p* < 0.01 vs. sham; #*p* < 0.05, ##*p* < 0.01 vs. ulinastatin.
